# A Proposed New Homœopathic Hospital

**Published:** 1888-12

**Authors:** 


### A PROPOSED NEW HOMŒOPATHIC HOSPITAL.

A meeting of homœopathic physicians was held at the office of Dr. J. A. Biegler, on South Clinton Street, Rochester, on Tuesday evening, at which Dr. Biegler presided, and Dr. Baker acted as Secretary. The result of the meeting was the adoption of the following sentiments :

WHEREAS, We, the members of the Rochester Hahnemannian Society, feeling the urgent need of a hospital where the principles of pure Homœopathy may be put in practice, and realizing that a proper time has come for the establishment of such an institution, therefore be it

*Resolved,* That this Society hereby assumes the responsibility of inaugurating such steps as will insure the speedy building and completion of a homœopathic hospital in Rochester, and to further such an undertaking the following Committee is hereby appointed to arrange a Board of Trustees for said hospital, and to transact such other business as may be necessary : J. A. Biegler, Chairman, Julius Schmitt, M. D., Allen B. Carr, M. D., R. C. Grant, M. D.

WHEREAS, We, the members of the Rochester Hahnemannian Society, who are in membership with the Monroe County Homœopathic Medical Society, fully believing in the rules of practice as given in the *Organon* of Samuel Hahnemann, the master, that the fundamental principles as therein given, viz., the law of similars, the totality of the symptoms, the single remedy, and the dynamic power of the drug, should be the sole foundation upon which we act in practice. And that further, that as legitimate Hahnemannian homœopathists we dissolve all the innovations which have been foisted upon Homœopathy by its false practitioners, and therefore we repudiate the mixing and alternating of medicines, and disapprove of all local and mechanical applications in non-surgical diseases, and

WHEREAS, The present active membership of the Monroe County Homœopathic Medical Society has heretofore maintained and does still continue a method of practice incompatible with the above principles, and has taken a further departure in assuming a hostile attitude toward the teachings of the founder of Homœopathy and his true followers, and compromising their professional principles by endeavoring to reconcile their practice with the teachings of the dominant school ; now, therefore, be it

*Resolved,* That we deem it a duty we owe to the public and to ourselves to withdraw from the Monroe County Homœopathic Society, and we hereby tender our resignations.

J. A. Biegler, M. D.,
R. A. Adams, M. D.,
Julius Schmitt, M. D.,
Allen B. Carr, M. D.,
Volney A. Hoard, M. D.,
R. C. Grant, M. D.,
W. G. Brownell, M. D.

Dr. Baker, the Secretary of the meeting, said: "The members of the Monroe County Society, whose names appear, have been thinking for some time of taking this step. They claim, as stated in the resolutions, that to alternate medicine or in any way deviate from the *Organon* of Hahnemann is wrong, and that no physician who does this can be a true homœopathist. The idea of building a hospital has perhaps hastened the action, as the members of our Society do not wish any one interested in it who does not follow the true homœopathic principles."

Dr. C. R. Sumner, on being interviewed, said: "I do not think the members of the Monroe County Homœopathic Society will be surprised at these resignations. The matter has been cooking for some time. The Hahnemannian Society was founded by several of this faction."

"Are their claims, as to following the true principles of Hahnemann, true in your opinion?" he was asked.

"Personally speaking, I feel that I am as good a follower of Hahnemann as they are. This faction is what we term 'high dilutionists.' They differ in the definition of Homœopathy from me. I hold that where a patient is ill beyond recovery that I have a right to use medicines which will relieve him and ease him from pain; so do many others of our school. That is one of the essential points of difference between us."

